# Review of Emotion Regulation Psychosocial Interventions for Children and Adolescents with ADHD

**DOI:** 10.3390/brainsci16060560

**Published:** 2026-05-25

**Authors:** Rosanna Breaux, Charity Majusiak, Annah R. Cash, Stephanie N. Pham, Michelle Le

**Affiliations:** Child Study Center, Department of Psychology, Virginia Tech, 460 Turner St. NW Suite 207, Blacksburg, VA 24060, USA; cmajusiak@vt.edu (C.M.); annahcash@vt.edu (A.R.C.); phamsn@vt.edu (S.N.P.); lmichelle@vt.edu (M.L.)

**Keywords:** attention-deficit/hyperactivity disorder, emotion dysregulation, treatment, transdiagnostic, families

## Abstract

Emotion dysregulation (ED), emotional responses that impair goal-directed behavior, have been argued to be a core characteristic of attention-deficit/hyperactivity disorder (ADHD), and are associated with functional impairment beyond the core symptoms of ADHD. Additionally, deficits in emotion regulation (ER), the ability to increase, decrease, or maintain the intensity, duration, and trajectory of emotions are common among children and adolescents with ADHD. Despite this, treatment efforts to improve such ER abilities and to decrease ED in youths with ADHD have been limited, particularly during the adolescent developmental period. Clinical knowledge was used to inform PsycInfo and Google Scholar searches to identify the relevant literature. This narrative review discusses transdiagnostic interventions (cognitive behavioral therapy, the Unified Protocol for Children/Adolescents, dialectical behavior therapy, acceptance and commitment therapy, and mindfulness-based interventions) that can be used to target ER in ADHD, and interventions for youths with ADHD that either have been found to improve ER outcomes or that directly target ER/ED for youths with ADHD and their families. We end our review by highlighting the gaps and limitations in the existing literature prior to drawing conclusions and making recommendations for future research in this important area.

## 1. Introduction

It is increasingly recognized that children and adolescents with attention-deficit/hyperactivity disorder (ADHD) frequently experience co-occurring emotional difficulties, posing significant functional challenges in addition to those posed by core symptoms of inattention and hyperactivity/impulsivity [1,2,3]. Deficits in emotion regulation (ER; i.e., the ability to increase, decrease, or maintain the intensity, duration, and trajectory of emotions [4,5]) have been well-supported as a potential mechanism contributing to emotional difficulties for youths with ADHD [6]. Emotion dysregulation (ED), a transdiagnostic phenomenon characterized by emotional responses that impair goal-directed behavior, is one of the most common reasons children and adolescents are referred for clinical services, including youths with ADHD [7,8]. ED is argued to be an impairing core feature of ADHD that is associated with functional challenges due to poorly modulated transitions between emotional states, and atypical allocation of attentional resources to emotional stimuli [6,9,10]. Specifically, children and adolescents with ADHD experience significant challenges with ER and ED, marked by increased temper outbursts, use of fewer adaptive self-regulation strategies, and more frequent and severe negative affect [11,12]. Although ED is often associated with insufficient regulation of negative emotions (e.g., anger, frustration, sadness), recent studies have highlighted that the dysregulation of positive emotions (e.g., exuberance) is also associated with functional impairments in those with ADHD (e.g., [13,14]). Despite this, limited psychosocial intervention research among youths with ADHD has specifically sought to target ER deficits and/or ED in this population. For example, a relatively recent systematic review and meta-analysis of ER psychosocial interventions for adolescents found only one study that involved adolescents with ADHD (see [15]).

## 2. Scope and Approach

Clinical knowledge was used to inform PsycInfo and Google Scholar searches to identify the relevant literature; search terms included the name of the intervention and ADHD, ER and ADHD intervention, or ED and ADHD intervention. Searches were independently conducted by three of the authors to ensure that relevant articles were included. Articles needed to be available in English, and involve interventions directly working with youths with ADHD and/or targeting ER/ED to be included in this review. We chose a narrative review over a systematic review considering the limited number of studies specifically targeting or measuring ER/ED outcomes among youths with ADHD. Table 1 provides an overview of the relevant psychosocial interventions, including citations for the studies utilizing the various interventions among youths with ADHD, and noting if ER and/or ED outcomes were included as primary or secondary outcomes.

In this narrative review, we start by discussing transdiagnostic psychosocial interventions that can be used to target ER deficits and/or ED in youths with ADHD. We then review psychosocial interventions for youths with ADHD that have been found to improve ER and then discuss the handful of interventions directly targeting ER/ED for youths with ADHD. We end by highlighting the limitations and gaps in the existing interventions and research and providing suggestions for future areas of research and intervention development/modification. This focus on psychosocial interventions specifically is being undertaken given other recent reviews and meta-analyses focused on pharmacological interventions for ED in ADHD [50,51,52].

## 3. Transdiagnostic Interventions That Can Be Used to Target ER in ADHD

Although standard ADHD treatments can reduce core symptoms and improve functioning, ER/ED concerns frequently persist or remain insufficiently addressed, particularly when negative affect, stress, or interpersonal conflict amplifies impulsive responding [53,54]. These observations have motivated interest in transdiagnostic interventions—treatments developed to target shared mechanisms across disorders that may be applied or adapted to address ER/ED within youths with ADHD. Importantly, transdiagnostic protocols vary in the degree to which ER is the explicit target, the developmental tailoring required for children/adolescents versus adults, and the extent to which efficacy data comes from ADHD-primary samples versus emotional disorder cohorts in which ADHD is represented as a comorbidity. Accordingly, the sections below summarize the key transdiagnostic approaches with the potential to improve ER/ED in pediatric ADHD—including cognitive behavioral therapy (CBT), the Unified Protocol for Children/Adolescents (UP-C/A), dialectical behavior therapy (DBT), acceptance and commitment therapy (ACT), and mindfulness-based interventions.

### 3.1. Cognitive Behavioral Therapy (CBT)

Despite originally being used for anxiety, depression, and obsessive–compulsive disorders, CBT [55] is a transdiagnostic, skills-based approach that can be leveraged to address ER/ED problems in youths with ADHD by targeting cognitive processes that intensify affect, reducing avoidant or emotion-driven behavioral patterns, and strengthening coping ability under affective load (e.g., problem-solving, cognitive reappraisal/reframing, relaxation, communication skills, behavioral strategies) [16,56,57]. In fact, there is even a book, Helping Children with ADHD: A CBT Guide for Practitioners, Parents, and Teachers [58] that is available and provides guidance on how to implement CBT techniques for parents and teachers, as well as guidance for practitioners working with parents and teachers on how to introduce and extend techniques into the home and classroom.

Individual CBT trials for adolescent ADHD have typically focused on ADHD impairment while also assessing ER symptoms as secondary outcomes [57]. For example, in a randomized controlled trial of CBT for adolescents with persistent ADHD symptoms despite medication use, CBT produced greater improvements than waitlist comparisons on blinded independent evaluator ratings of ADHD symptom severity as well as global functioning based on both parent and adolescent report [21]. Other adolescent CBT adaptations similarly incorporate emotion-focused components (e.g., anger/frustration, coping with stress, cognitive restructuring) alongside core organizational/time-management and behavioral activation elements [16,17,59,60]. Complementing efficacy trials, feasibility work suggests that group-based CBT programs for adolescents with ADHD that include ER-relevant content (e.g., frustration/anger management) are generally acceptable to older adolescents with ADHD and can be implemented with good attendance and low dropout in outpatient settings [61].

Importantly, the specific evidence base for CBT effects on ER/ED outcomes in youths with ADHD remains smaller and more heterogeneous than the adult literature. For example, a systematic review focused on psychosocial interventions for children with ADHD and ED concluded that available randomized controlled trials, including CBT and CBT-adjacent approaches, are promising for irritability/severe emotional behavior problems but are limited by variability in targets/outcomes and relatively sparse randomized evidence [62]. In tandem with this, a recent meta-analysis of non-pharmacological interventions for emotional symptoms in ADHD found that youth emotional-symptom improvements were most consistently supported for relationship- and social-focused interventions (e.g., parent training and social skills training), whereas CBT showed clearer benefits for emotional symptoms in adults, underscoring that CBT for ER in youths with ADHD remains comparatively underpowered and likely dependent on developmental fit and treatment format [63].

Consistent with these broader patterns, at least one adolescent group/CBT randomized controlled trial reported no incremental benefits over comparison groups on emotional symptoms [56], highlighting the practical point that CBT protocols may include ER content without ER being consistently positioned as a primary treatment target or primary endpoint. Notably, even when incremental effects are not consistently observed on secondary emotional symptom outcomes, adolescents may still report high satisfaction with group-CBT formats and view them as feasible and helpful, which has implications for engagement and dissemination [61]. Finally, clinical reviews of CBT for adolescent ADHD emphasize that therapeutic configuration and co-occurring conditions associated with ED may matter for ER-relevant gains, such as considering parental involvement differently for externalizing versus internalizing comorbidity; however, these moderators remain incompletely tested [16]. Notably, to our knowledge, no study on CBT for youths with ADHD [12,16,17,21,57] has examined ER or ED as a primary or secondary outcome, though related constructs, like self-control of impulsivity, have been examined [20]. Overall, CBT for youths with ADHD frequently incorporates ER-relevant skills, particularly frustration/anger-management strategies, yet more targeted research is still needed to clarify the optimal CBT format (i.e., individual vs. group), parent involvement, and developmental tailoring to address ER/ED concerns in this population.

### 3.2. Unified Protocol for Children/Adolescents (UP-C/UP-A)

The UP-C/A [64,65] is an emotion-focused CBT treatment adapted to target shared, transdiagnostic ER processes across emotional disorders (anxiety, depression) and related problems in children and adolescents. In UP-C/A, these targets are operationalized via a unified set of CBT strategies that are targeted across multiple emotion domains, and are typically delivered in a structured format with caregiver involvement to support the generalization of skills [64]. Importantly, UP-C can improve ER-relevant processes alongside symptom reduction [65,66,67]. For example, in an initial open trial study with children with anxiety disorders and with and without comorbid symptoms of depression, from pre- to post-treatment, medium-to-large improvements were shown in anxiety disorder severity and symptoms of depression; 78% of children did not meet the criteria for any anxiety disorder at post-treatment [66]. More recently, in a pilot randomized controlled trial comparing UP-C to an active anxiety-focused intervention, both conditions reduced anxiety, while UP-C generated relatively greater gains on several ER-related variables (e.g., lower parent-rated sadness dysregulation and higher child-rated cognitive reappraisal; [67]). Systematic reviews and meta-analyses support the efficacy of the UP-C/A framework across emotional disorders and related transdiagnostic mechanisms, providing broader evidence for this ER-focused transdiagnostic model [68,69].

However, UP-C/A has not been directly tested in samples recruited for ADHD, despite ADHD being represented as a comorbid condition in the existing studies of UP-C/A for youths with emotional disorders [70], and the high rates of co-occurrence between ADHD, anxiety, and depression (see [71,72]). For example, in a Portuguese UP-C feasibility study of children with primarily emotional disorders, 18.8% of the enrolled sample had comorbid ADHD, but the study also notes that youths were excluded when ADHD or oppositional defiant disorder was the principal diagnosis [22], underscoring that the existing UP-C/A trials have generally not been designed to evaluate efficacy for ER/ED specifically within ADHD samples.

### 3.3. Dialectical Behavioral Therapy (DBT)

Although CBT for youths with ADHD often includes ER-relevant skills, ER is inconsistently emphasized as the central treatment target and, as mentioned previously, has never been evaluated for ER/ED outcomes in this population. By contrast, DBT is explicitly designed to address ER/ED through the repeated practice of skills such as mindfulness, acceptance, behavioral chain analysis, ER, and interpersonal effectiveness [73]. These methods are intended to reduce emotion-driven actions and to strengthen in-the-moment regulation. DBT Skills Training has only recently been used among adolescents with ADHD [23,28,29], despite being commonly used for adults with ADHD (see [24] for a review). Specifically, Landin and colleagues (2025) evaluated the feasibility and preliminary efficacy of a 10-week DBT skills training group among 10 adolescents with ADHD, and found pre- and post-improvements across primary and secondary outcomes, including ADHD symptoms, executive function (which includes ED as one of the five domains in the outcome), internalizing symptoms, and quality of life [23]. Notably, only 8 of the 10 participants completed the treatment, though attendance and treatment satisfaction were high for those who did complete, and this was a pilot study without a control group.

Further evidence for use of DBT among adolescents with ADHD comes from the Structured Skills Training Group (SSTG [26]). SSTG is an age-adapted, manualized, DBT-based, group intervention originally developed for adults with ADHD that has been modified for adolescents (e.g., simplified language, more practical exercises; [26]). Twenty adolescents with ADHD who participated in the SSTG completed interviews following treatment, where they described emotional and behavioral changes, such as higher self-esteem, fewer inter-personal conflicts, and improved concentration [26]. Qualitative data also indicate that adolescents often experience DBT-based skills groups as improving emotional control and coping (e.g., using mindfulness and acceptance skills), even while noting that skills can be hard to apply consistently in daily life [27]. In a large multi-site randomized controlled trial of adolescents with ADHD (ages 15–18; *n* = 184 randomized), the SSTG was compared to an active psychoeducational control; the trial found no significant between-group differences for ADHD symptoms, behavioral/emotional problems, functional impairment, or health-related outcomes, although both conditions were rated as acceptable/helpful (e.g., increased ADHD knowledge; improved ability to manage ADHD-related problems; willingness to recommend [28]). However, recent secondary analyses examining moderation of treatment outcomes suggest potential heterogeneity in response, such that youths with ADHD displaying greater baseline ED, hyperactivity/impulsivity, and related externalizing features (i.e., conduct problems) may show comparatively better outcomes with the SSTG [29], highlighting a potential for DBT-based skills training to be recommended only for some youths with ADHD (i.e., those with combined presentation and elevated ED). This is an important area for future research to further explore to assess which youths with ADHD may benefit most from DBT.

Importantly, DBT has also been extended downward for use with children (DBT-C) with ADHD and related concerns, like disruptive mood dysregulation disorder and impulsive aggression [25,74]. DBT was first extended for use with children with disruptive mood dysregulation disorder, which has a high co-occurrence with ADHD, but ER/ED outcomes were not directly assessed [74]. More recent evidence from a randomized controlled trial with 66 children with ADHD and impulsive aggression, found significant reductions in the severity of their impulsive aggression and ER difficulties following the completion of DBT-C skills training, relative to an ADHD medication and psychoeducation control group [25]. Interestingly, in this study, two DBT-C skills training groups were conducted, with one being for children (ages 6–9 years) and one for early adolescents (ages 10–13 years), highlighting the need for interventions to focus on relevant emotional development. Further, it has been argued that DBT-C could be integrated into behavioral parent training (BPT) interventions, a first-line treatment for youths with ADHD and externalizing concerns [75], highlighting an important area for future research to explore. Specifically, they review three ways DBT and BPT can be integrated, as follows: (1) DBT for adults tailored to parents that integrates parenting strategies [76,77]; (2) DBT trials that have BPT incorporated into the intervention [74,78]; (3) BPT that incorporates the components of DBT skills [79].

### 3.4. Acceptance and Commitment Therapy (ACT)

Whereas CBT and DBT primarily build ER abilities through teaching discrete skills (e.g., cognitive reappraisal, distress tolerance), ACT [80] conceptualizes ED as a problem of psychological inflexibility (e.g., experiential avoidance, cognitive fusion) and aims to increase the individuals’ capacity to notice and tolerate difficult internal experiences while engaging in goal-directed behavior consistent with their values. Beyond teaching strategies to downshift arousal, ACT emphasizes willingness to experience uncomfortable emotions and thoughts while still engaging in goal-directed behavior, an approach that may address ADHD-related emotion-driven impulsivity and avoidance under stress [32,80]. Conceptually, ACT’s core processes (i.e., acceptance, cognitive defusion, present-moment awareness, values clarification, committed action) map well onto common ER vulnerabilities in ADHD (e.g., rapid affective escalation, low distress tolerance, and impulsive ‘escape’ behaviors under negative affect/cognitive load). As youth move into adolescence and emotional experiences become more complex, interventions that support adaptive responding without requiring immediate emotion change may be particularly useful; ACT provides this via acceptance, cognitive defusion, and values-guided action [80].

However, in youths with ADHD, the evidence base for ACT remains quite small, and the outcomes assessed are often ADHD symptoms or functional proxies rather than ER/ED. A recent scoping review of ACT for ADHD identified only a handful of studies with children, adolescents, or adults (*n* = 6), and concluded that the findings, while promising, are still largely preliminary, highlighting small sample sizes and limited methodologically rigorous trials [32]. For example, a pilot study of a program based on ACT for Kids [81] with nine adolescents aged 11–15 years with ADHD, found significant reductions based on a reliable change index in five of the seven youths who completed the treatment [33]. Similarly, two open-label Italian studies evaluated the same 9-month (26-session) ACT-based group child training delivered after ACT-based parent training, but with different outcomes [34,35]. In the clinical effects study (ages 8–13; *n* = 31), the primary outcomes were ADHD behavioral symptom ratings and clinician severity on the Clinical Global Impression scale, and the authors found significant pre- and post-improvements on multiple parent and clinician symptom ratings [35]. They also found significant improvements in the Conners’ Parent Rating Scales-Revised Global Index, which consists of Restless–Impulsive and Emotional Lability, providing some evidence for improvements in ED following ACT, but this needs to be replicated in other studies assessing ER/ED directly and independently of other related outcomes. By contrast, in the cognitive domain study (ages 8–13; *n* = 36), the primary outcome was cognitive performance assessed via a battery of computerized tasks, with the findings indicating no significant improvements in cognitive measures [82]. Overall, while there is some evidence of improvement in ADHD symptoms and quality of life, following ACT either with the youths alone or with the parents and then the youths, more research is needed to examine the benefits of ACT for ER deficits and/or ED directly.

### 3.5. Mindfulness Interventions

Mindfulness-based interventions train sustained attention and nonjudgmental awareness of internal experiences (e.g., thoughts, emotions, and bodily sensations). In theory, these practices support ER by improving early detection of emotional cues, decreasing automatic/reactive responding, and strengthening the capacity to “pause” and select a response rather than act impulsively—processes that may be especially relevant in ADHD, where difficulty inhibiting prepotent responses can contribute to impulsive and reactive behavior [82,83,84]. A recent systematic review and meta-analysis of randomized controlled trials in children and adolescents with ADHD found that mindfulness-based interventions generated reductions in ADHD symptoms, with comparatively larger effects observed among older youths/adolescents, and also noted benefits in broader domains commonly intertwined with ER/ED and family functioning (e.g., internalizing and externalizing symptoms, mindfulness skills, and parenting stress; [36]). Consistent with this, broader meta-analytic work spanning age groups supports improvements related to mindfulness-based interventions in ADHD symptoms relative to waitlist/control conditions, while underscoring meaningful heterogeneity across outcomes and variability in study quality [37].

Based on these systematic reviews and meta-analyses, to our knowledge, only one study directly assessing ER or ED outcomes among mindfulness interventions for youths with ADHD has been conducted [38]. This study, which was conducted with adolescent females aged 13–15 years with elevated ADHD symptoms were randomly assigned to either a mindfulness treatment or a waitlist control group, had findings indicating that adolescents assigned to the mindfulness treatment displayed significantly lower ED post-treatment relative to the control group [38].

Within the studies focused on youths with ADHD, the studies have commonly implemented mindfulness in family-inclusive formats (e.g., child skills practice paired with mindful parenting components), reflecting the clinical rationale that caregiver scaffolding may facilitate generalization of self-regulation skills to everyday contexts [16,83]. For example, MYmind—a structured family-based mindfulness program that includes parallel group sessions for youths and parents (i.e., mindful parenting) with between-session practice—has been developed [39]. Recent randomized trials of MYmind have compared this mindfulness program to an evidence-based active control (i.e., CBT), helping to distinguish mindfulness-specific benefits from nonspecific influences, such as group support, therapist contact, and treatment expectancy [40]. They found no significant difference for both primary and secondary treatment outcomes between the two arms at either time point, though found both to significantly improve the primary outcome of attention and secondary outcomes of attention, ADHD symptoms, disruptive behavior, mindfulness, well-being, and executive function (which includes ED as one of the five domains in the total score).

Despite these encouraging findings and clear mechanistic fit with ER/ED targets, ER/ED outcomes remain inconsistently operationalized and rarely directly measured across mindfulness studies of youths with ADHD, with the findings often being inferred from changes in internalizing/externalizing symptoms rather than assessed with dedicated measures of ED, emotion lability/reactivity, ER strategy use (e.g., reappraisal/acceptance), or behavioral indices of frustration tolerance—highlighting a key next step for establishing mindfulness-based interventions as a more definitively targeted ER/ED intervention in youths with ADHD [36].

## 4. Interventions for Youths with ADHD That Have Components Targeting ER/ED

Beyond these transdiagnostic interventions, there are a handful of interventions specific to youths with ADHD that include a component or primary focus of targeting ER or ED. Several of these interventions focus primarily on social and academic functioning, but include a self-regulation/mindfulness component: the Challenging Horizons After-School Program (CHP; [85]) and Child Life and Attention Skills (CLAS; [86])/Collaborative Life Skills (CLS; [43]). The other interventions specifically targeting ER for youths with ADHD fall into the following two primary categories: (1) early interventions that work directly with parents to improve emotion socialization and other parenting practices for parents of preschoolers with ADHD [44,45,87] or school-age children [46]; and (2) interventions working with the child/adolescent to build ER skills, either alone [47,48] or in combination with caregiver involvement [30,49].

### 4.1. The Challenging Horizons After-School Program (CHP)

CHP was developed for adolescents with ADHD in middle school, and was then extended to high school students [85,88]. CHP generally involves students staying after school to participate in group and individual interventions targeting both the social and academic challenges these youths may experience [85]. CHP is held 2–4 days per week, for about 2 h each day, throughout the academic year, and indirectly targets ER through structured interpersonal skills groups utilizing problem solving and goal setting during social activities [85]. Within these groups, adolescents reflect on past social interactions and plan future responses that align with their personal social goals [42]. Early pilot studies and randomized control trials of CHP demonstrated significant improvements in academic functioning, organizational skills, and social functioning among middle and high school adolescents with ADHD [85,88]. Despite not directly targeting ER/ED, recent evidence indicates significant small-to-moderate improvements in parent-rated ED for high school students with ADHD following completion of CHP relative to community care [42].

### 4.2. Child Life and Attention Skills (CLAS)/Collaborative Life Skills (CLS) Program

The CLAS Program is a multi-component, psychosocial treatment intended for 2nd–5th grade youths with ADHD, Predominantly Inattentive Presentation (ADHD-I) [86]. CLAS is tailored to address multiple domains of functioning through ten group-based parent training sessions, ten group-based child life skills training sessions, and teacher consultation with classroom intervention supports. In essence, CLAS focuses on the unique challenges children with ADHD-I may face across multiple treatment settings. The utilization of positive reinforcement and the implementation of supportive behavioral strategies may reduce ED in these youths, such that improvements in daily functioning and reductions in impairments across home and school contexts may indirectly support more adaptive emotional functioning. CLS, a school-based adaptation of CLAS, was later developed to translate these intervention components into school systems [43]. The findings from a CLAS pilot study and a subsequent randomized control trial demonstrated significant treatment effects for organizational, social, and inattention impairments [86,89]. It will be important for future work to explore whether CLAS/CLS can improve ER and ED outcomes directly.

### 4.3. Parent–Child Interaction Therapy with Parent Emotion Coaching (PCIT-ECo)

Considering that Parent–Child Interaction Therapy (PCIT) is a gold standard intervention for youths with disruptive behaviors [90], efforts to adapt PCIT for ED in preschoolers with ADHD have been made. One such adaptation is PCIT-ECo, which incorporates the integration of parent emotion coaching skills and parents’ implementation of these ER abilities during live parent–child interactions for preschoolers with ADHD [44]. PCIT-ECo builds upon PCIT-Emotion Development [91,92], which was originally developed to treat preschool depression through the emphasis of emotion recognition and relaxation skills. In adapting PCIT-ED for preschoolers with ADHD, PCIT-ECo reduces the focus on direct child instruction in emotion identification and prioritizes coaching caregivers in emotion socialization and modeling prosocial ER strategies across five sessions [44]. In a pilot study examining PCIT-ECo, improvements were observed in child ER, externalizing problems, and overall impairment following treatment and a 6-month follow-up [44].

### 4.4. Parenting Your Hyperactive Preschooler

Like PCIT-ECo, the Parenting Your Hyperactive Preschooler program is designed for caregivers with preschoolers with ADHD [87] through structured BPT. However, Parenting Your Hyperactive Preschooler is based primarily on behavioral principles and emphasizes parent management of disruptive and hyperactive behavior. In addition to the behavioral training, Parenting Your Hyperactive Preschooler incorporates caregiver emotion socialization in sessions 9 through 14. Session 9 focuses specifically on psychoeducation for ER, while subsequent sessions train caregivers in adaptive emotion coaching strategies, including emotion identification and labeling, managing negative emotions, and boosting positive emotions. A randomized control trial of Parenting Your Hyperactive Preschooler found that parents who participated in the program reported improvements in their children’s emotional lability/negativity [45]. Given the program’s emphasis on teaching caregivers’ supportive emotion socialization skills (e.g., validating and labeling children’s negative affect), these findings suggest that strengthening parents’ responses to children’s emotion may contribute to reductions in emotional lability [45].

### 4.5. Incorporating ED into BPT

BPT has been established as an efficacious evidence-based intervention for school-aged children with ADHD [88], with substantial evidence demonstrating reductions in disruptive behaviors and ADHD symptoms following treatment. However, traditional BPT programs primarily focus on behavior management strategies (e.g., reinforcement, consistent consequences) rather than directly targeting children’s emotional processes. Given that traditional BPT approaches do not fully address parents’ emotion regulation skills or their abilities to support children’s emotional experiences, researchers have increasingly emphasized the importance of integrating emotion-focused components into BPT to better address the emotional processes underlying behavioral difficulties in ADHD. Addressing this gap, Diaz (2025) developed a behavioral framework for incorporating ED into BPT for school-aged children [46]. This framework integrates an ED-focused psychoeducation component that helps parents understand the physiological, emotional, and behavioral processes underlying children’s dysregulated emotional responses. Specifically, the framework emphasizes aligning parenting strategies with children’s emotional states and strengthening caregivers’ emotion coaching and de-escalation skills within the context of existing BPT programs. Preliminary pilot findings suggest that integrating this framework within BPT may improve parents’ ability to manage episodes of ED and reduce the frequency, intensity, and duration of children’s dysregulated emotional states [46]. 

### 4.6. Managing Frustration for Children (MFC)

In contrast to prior interventions that primarily target parenting practices, the MFC intervention focuses directly on improving children’s ER skills [47]. MFC is a 12-session group-based intervention designed for children with ADHD that targets frustration tolerance and dysregulated emotion reactivity. The program teaches children a range of cognitive, behavioral, and physiological coping strategies to help them manage emotional distress. Early sessions focus on providing psychoeducation about ER, while introducing problem-solving strategies to help children inhibit impulsive emotional responding. Subsequent sessions emphasize the development and practice of coping strategies for regulating emotional distress. Midway through MFC, parents attend a psychoeducation session in which clinicians review the skills taught in the child group sessions and provide guidance on supporting children’s emotion regulation at home [47]. A pilot study of MFC found significant reductions in internalizing, externalizing, and ED problems [47].

### 4.7. Emotion Regulation Skills (ER-SKILLS) 

Like MFC, ER-SKILLS [48] seeks to target ED, but in adolescents with ADHD, through eight sessions including techniques and exercises from CBT [55], DBT [73], and ACT [80]. ER-SKILLS is theoretically based on the Extended Process Model of ER [93]. Specifically, the first three sessions focus on emotion awareness, emotion clarity, and acceptance of emotions; sessions 4–6 focus on various ER strategies, session 7 focuses on flexibility in strategy choice, and session 8 focuses on sustained positive change and goal setting. ER-SKILLS has been evaluated using a multiple-baseline single-case experimental study with seven girls aged 13–17 years with ADHD with weekly self- and caregiver-ratings for ED [48]. They found that four of the seven girls reported statistically lower ED after intervention; however, six adolescents and four caregivers reported improvement before the intervention, limiting conclusions from being drawn regarding intervention effects.

### 4.8. Regulating Emotions Like an eXpert (RELAX)

The RELAX intervention was developed to improve ED and interpersonal conflict for adolescents with ADHD and their caregivers [30]. RELAX focuses on teaching caregivers to reinforce ER and communication skills and to respond more effectively to adolescents’ emotional experiences. RELAX has been delivered in-person and via telehealth, and consists of 8 weekly 90-min sessions and two booster sessions 1- and 6-months post-intervention [30,49]. During the first 60 min of each group, parents and adolescents meet separately for psychoeducational and skills-based groups that involve reflective and discussion activities and role plays. Within RELAX, caregivers are provided with ER and communication skills one week prior to their adolescents to help the parents understand and support the ER strategies their adolescents will learn later, prior to moving to simultaneously teaching and practicing conflict management strategies. Two pilot studies of RELAX found significant reductions in adolescent ED and improvements in adolescents’ ER abilities, as well as increases in supportive parental emotion socialization practices following treatment [30,49]; however, these findings need to be tempered given the lack of a control group. Currently, RELAX is being evaluated through a randomized controlled trial (National Institute of Mental Health, K01 MH136333-01A1).

## 5. Discussion

This narrative review discussed the various transdiagnostic and ADHD-specific psychosocial intervention approaches for improving ER and reducing ED among children and adolescents with ADHD. It extends recent reviews and meta-analyses focused on pharmacological interventions for ED in ADHD [50,51,52] by suggesting the promise of both ADHD-specific and transdiagnostic psychosocial interventions to improve ER skills and to reduce ED in this at-risk population. It will be critical for future intervention efforts to consider the combined or sequenced use of psychosocial interventions with pharmacological interventions for targeting ED in youths with ADHD. Given the significant side effects present among many of the effective treatments (e.g., antipsychotics, mood stabilizers), it may be ideal to start with psychosocial interventions prior to moving to medication, as has been found for the sequencing of behavioral parent training and stimulant medications [37,78,92].

Consistent with transdiagnostic interventions like CBT and DBT being focused on negative emotions, the reviewed interventions heavily focused on ED of negative emotions among youths with ADHD. While this makes sense from the standpoint of negative emotions generally being less acceptable and more impairing than positive emotions, given the growing recognition of positive emotion ED being associated with functional impairments in those with ADHD (e.g., [13,94]), it will be critical for ongoing and future research efforts to focus on this aspect of ED as well. Of note, within our own work implementing the RELAX intervention with families of adolescents with ADHD, this is often performed informally through the parent–adolescent discussions during the last 30 min of sessions each week, where skills are applied to that specific child and family. Similarly, this could be extended to other transdiagnostic and ADHD-specific intervention. For example, using a mindfulness-based program, one can train themselves to fully savor and appreciate positive experiences, notice joy without immediately clinging to it or becoming overwhelmed, and cultivate gratitude in their lives. In fact, there is research supporting that mindfulness training can promote positive emotions [95]. Additionally, when discussing coping skills, clinicians often discuss how these can be used proactively as well as to manage the distraction that can come from excitement or anticipation regarding a positive, upcoming event during the adolescent group sessions. As reviewed in the next two sections, the evidence from both transdiagnostic and ADHD-specific interventions is encouraging. Despite this promise, as highlighted throughout this discussion, there are gaps in the existing intervention literature and important areas for future research to further refine and improve intervention efficacy.

### 5.1. Utilization of Transdiagnostic Approaches to Improve ER/ED in Youths with ADHD

To date, transdiagnostic approaches that can be applied to target ER/ED in youths with ADHD have focused on school-aged children and adolescents (i.e., ages 6+ years). Given that ER first develops during the preschool years, and that CBT for anxiety and post-traumatic stress disorder have been utilized with success during these early developmental periods [96,97], this could be important for future research to explore as a preventative means for children with ADHD. It is important to highlight that, despite more recent studies specifically utilizing these approaches for youths with ADHD, these interventions have generally focused on internalizing disorder populations, and either did not report on co-occurring ADHD diagnoses or excluded such diagnoses, limiting the number of studies to date. Further, it is possible that families of children and adolescents with ADHD may be harder to engage in treatment [98,99] or to have family involvement due to parent mental health and executive functioning concerns [99,100].

Across the existing transdiagnostic approaches, DBT and UP-C/A most explicitly conceptualize treatment around ER mechanisms, whereas CBT and mindfulness typically influence ER through broader skills acquisition and attention/awareness processes. For ACT, it is theorized to impact ER indirectly via psychological flexibility. In terms of the ADHD-specific evidence of improvements in ER/ED outcomes in youths for transdiagnostic treatments, mindfulness-based programs and adolescent CBT currently represent the largest ADHD-targeted body of literature, although ER/ED outcomes are commonly not assessed or treated as secondary endpoints rather than primary mechanisms or targets [36,56,57]. DBT skills training is a particularly relevant emerging option for children and adolescents with ADHD, given its direct ER skills emphasis, though effects have been modest and may vary by baseline ED or ADHD/externalizing symptom severity [20,25,29]. It is critical that future research utilize person-centered analyses and/or continue to examine moderators of treatment outcomes rather than only assessing pre- and post-outcomes on average.

By contrast, UP-C/UP-A and ACT appear best characterized as transdiagnostic frameworks with high potential for targeting ER/ED that still require more rigorous evaluation in samples where ADHD is the primary presenting condition and ER/ED is an explicit treatment target, particularly given that many UP-C/A and ACT trials have been conducted in samples with emotional disorders, with comorbid ADHD rather than primarily ADHD cohorts [22,32]. We encourage researchers and clinicians utilizing these various transdiagnostic intervention approaches to more systematically assess the benefit of the interventions on both ER skill acquisition (i.e., utilization of specific ER strategies) and reductions in ED.

### 5.2. Utilization of ADHD-Specific Approaches to Improve ER/ED in Youths with ADHD

Unlike the transdiagnostic intervention approaches, many of the ADHD-specific interventions have focused on preschool-aged children with ADHD through parenting interventions. Specifically, both PCIT-ECo [44] and Parenting Your Hyperactive Preschooler [45,87] focus on psychoeducation around ER and teaching emotion recognition and relaxation skills, in addition to focusing on the parenting practices in general that are common in BPT/PCIT as well as emotion-focused parenting practices (i.e., emotion coaching, emotion socialization). This incorporation of sessions targeting emotional development and ER/ED specifically into BPT was also utilized with school-aged children with ADHD [46]. Interestingly, for school-aged children and adolescents, there was a split for both developmental periods, with one intervention approach involving parents for each [46,49], and one working only with the child for each [47,48]. Given that all of these were pilot studies, it will be critical to formally assess whether having parent involvement leads to better outcomes utilizing controlled study designs. Encouragingly, all but two of the ADHD-specific interventions reviewed have at least one peer-reviewed manuscript that supports their benefit in addressing ER/ED among youths with ADHD [30,42,44,45,47,48,49], including through a 6-month follow-up period [44]. Given that most of these were pilot studies and only focused on pre- and post-treatment gains, more research examining long-term ER and ED outcomes is critically needed. Additionally, given the findings that CHP [42], an intervention focused on social and academic challenges, could improve ED for adolescents with ADHD, we encourage researchers conducting other ADHD-specific interventions (e.g., Organizational Skills Training, Supporting Teens’ Autonomy Daily) to assess whether those intervention can result in secondary improvements in ER/ED.

### 5.3. Importance of Including Parents When Targeting Youth ER/ED

It has been previously argued that youth self-regulation interventions need to include teaching parents ER skills in addition to the child [101], and that parent ER should be included as a target of child mental health treatment generally [102]. Consistent with this, many of the studies that have been successful in improving ER/ED among youths with ADHD have taken a family-focused approach [16,19,30,83]. For example, some of the interventions ran parallel parent and youth groups so parents could learn the same ER skills and receive psychoeducation about ER and mindfulness/coping strategies [22,30,39].

Given the high levels of psychopathology and ED present among the caregivers of youths with ADHD (see [103]), it makes sense from an emotion socialization standpoint [104] that targeting caregiver ER/ED is just as important as targeting ER/ED in youths. Specifically, by improving caregiver regulation, they are then better able to both model appropriate affective and regulatory displays and to support their child in utilizing ER skills. It is possible that sequencing treatments may also be an effective approach. For example, starting with a psychosocial intervention or BPT for the parent while starting pharmacological intervention with the child may create an environment in which the child and family is better able to then receive child-focused ED skills, given the significant and moderate reductions found for pharmacological treatments (e.g., guanfacine, atomoxetine) in reducing ED severity [50]. Such sequencing approaches have been previously explored among the families of youths with ADHD [94,105,106,107], as well as for medication and some of the discussed transdiagnostic approaches (e.g., [108]); however, to our knowledge, these have not specifically explored ER/ED as outcomes. Further, empirically evaluating the short- and long-term benefits and cost-effectiveness of such combined or sequenced ER intervention approaches in the families of youths with ADHD is an important area for future research to explore.

### 5.4. Assessing and Improving ER/ED in Daily Life

In addition to encouraging more research with both transdiagnostic and ADHD-specific psychosocial interventions that assess the short- and long-term benefits for ER/ED, as discussed above, one of the major limitations of the existing research is the inability to assess how learned skills are applied in real-world contexts. There has been growing interest in ecological momentary assessment and single-case experimental design to assess emotion regulation skill use during treatment (see [109]), including the use of digital technologies to assess the more dynamic nature of ER processes (see [110]). This is critical to assess how state and context/environmental factors may impact the youth’s ED and use of ER strategies in real time. Intervention research with families of youths with ADHD should consider utilizing such digital tools as smartphone apps, virtual reality, and physical activity approaches to assess how ER looks in daily life and to explore if such tools may also be used to help improve ER in youths with ADHD. In our own ongoing work, we are utilizing ecological momentary intervention to support skill use in both caregivers and youths during and following the completion of a psychosocial intervention targeting ED in adolescents with ADHD with the hope that this can improve both short- and long-term outcomes.

A final challenge that emerged in writing this review regards the significant heterogeneity in how ER/ED is measured and targeted by interventions. Some of this reflects developmental differences in emotional development for young children vs. adolescents [111], but it also reflects various theories of ER (e.g., [93,112,113]) and the plethora of ER and ED measures (e.g., respiratory sinus arrythmia, informant–report questionnaires). In order for meaningful conclusions to be drawn across intervention studies, it would be helpful for intervention researchers to include measures of both ER skills/strategies (e.g., Cognitive Emotion Regulation Questionnaire, Emotion Regulation Questionnaire for Children and Adolescents) and ED (e.g., Emotion Regulation Checklist, Difficulties with Emotion Regulation Questionnaire). Ideally, mixed-methods could be utilized, where informant–report measures are supported by observational and/or physiological data; however, such measures, while related, often contribute meaningfully distinct results (e.g., [114]), particularly given that observational data often involves interactions with parents and thus could be more reflective of co-regulation or the influence of parenting practices than self-regulation. However, such mixed-method approaches are uncommon. A recent systematic review found that only 25% of studies used more than one measure of ER in samples of autistic individuals [115]. Similarly, given the need to assess the benefit of including parents in such interventions for youths with ADHD, including a measure of parent ER and ED would also be worthwhile.

## 6. Conclusions

This review highlights the growing interest over the last two decades in exploring both transdiagnostic and ADHD-specific psychosocial interventions to target ER/ED among youths with ADHD. Although this body of work is promising, much of the research on ADHD-specific interventions has been limited to small pilot studies, and many of the transdiagnostic approaches have not directly recruited youths with ADHD or assessed ER/ED outcomes. In addition to encouraging further research on both treatment approaches, including studies examining short- and long-term outcomes, we highlight the need for future research to explore the potential added benefits of involving parents in treatment for children and adolescents with ADHD, as well as considerations related to treatment sequencing. Finally, we underscore the importance of continuing to move the field toward a common language for defining and measuring ER skills and ED, using multi-method assessment approaches, and considering how these constructs can be assessed in daily life rather than relying solely on retrospective informant–report measures.

## Figures and Tables

**Table 1:** Summary of Interventions Targeting ER/ED for Use with Children and Adolescents with ADHD.

Intervention Name	Intervention Key Components	Age Ranges	Number of Sessions	Studies with Youths with ADHD	ER/ED as a Primary or Secondary Outcome
**Cognitive** **Behavioral** **Therapy (CBT)**	Targets cognitive processes, reducing avoidant/emotion-driven behavioral patterns, and strengthens coping abilities to manage strong emotions.	3–18 years	12–16 sessions	[12,16,17,18,19,20,21]	Not assessed
Unified Protocol–Children/ Adolescents (UP-C/A)	Emotion-focused CBT Psychoeducation and CBT strategies to reduce anxiety/depression and to promote ER Parents participate in all sessions to learn same skills as child and to learn emotional parenting behaviors.	7–13 years (UP-C), 13–18 years (UP-A)	15 sessions	[22] (18.8% had an ADHD diagnosis)	Not assessed
**Dialectical** **Behavioral** **Therapy (DBT)**	Targets intense emotions, reduction of self-harm behavior, and improvement of relationships	6–18 years	10–14 sessions	[23,24]	Secondary
DBT Skills Training Program for Children (DBT-C) with ADHD and impulsive aggression	Focuses on and provides opportunities to practice ER, mindfulness, interpersonal effectiveness, distress tolerance, and parenting skills in group therapy format using role play, games, and relevant examples Gives tokens to children who successfully implemented the skill that week	6–13 years	12 sessions	[25]	Primary
Structured Skills Training Group (SSTG)	Adapted DBT intervention for adolescents that includes visual materials and active/experiential exercises (e.g., role play, discussions) Themed sessions with discussions and exercises to practice mindfulness, acceptance, and behavioral analysis, Workbook on DBT skills and PowerPoint presentations in session	15–18 years	14 sessions	[26,27,28,29]	Not Assessed or Moderator of Treatment Outcomes
**Acceptance and** **Commitment** **Therapy (ACT)**	Conceptualizes ED as problem of psychological inflexibility Aims to increase individual’s capacity to notice and tolerate difficult experiences Engage in goal-directed behavior consistent with values	8–15 years	Varies (10–26 sessions)	[30,31,32,33,34,35]	Not assessed, except combined with Restless– Impulsive symptoms
**Mindfulness-Based** **Interventions**	Train sustained attention and nonjudgmental awareness of internal experiences (i.e., thoughts, emotions, sounds, bodily sensations) Exercises include walking meditation, sitting mediation, mindful eating, mindful movements, and body scan	5–18 years	8–20 sessions	[36,37] (review/meta-analysis); [38] only study to include ER/ED as outcome	Not assessed or Primary
MYmind	Structured family-based mindfulness program for youths and parents simultaneously	8–12 years	8	[39,40]	Secondary
**Challenging** **Horizons** **After-School** **Program (CHP)**	After school program involving group and individual interventions for social and academic challenges Targets ER through structured interpersonal skills groups via problem solving and goal setting	10–13 years	2–4 days/week, for an academic year	[41,42]	Secondary
**Child Life and Attention Skills (CLAS)**	Addresses multiple domains of functioning impaired by ADHD-I: − Group-based parent training − Group-based child life skills training (independence and social skills) − Teacher consultation (classroom management strategies) Parent and child met together at end of group sessions to review “skill of the week”	7–11 years	10 parent groups; Up to 6 family meetings; 10 child groups; Up to 6 teacher consults	[41]	Not assessed
**Collaborative Life Skills (CLS)**	Adaptation of CLAS to address multiple domains of functioning impaired by generalized ADHD across multiple settings 3 core components: Classroom (school–home daily report card, homework plan, classroom accommodations) Parent (traditional parent training program skills) Child (targeting social functioning and independence)	2nd–5th grade (*M*age = 8.3)	2–3 meetings with parent, student, and teacher; 10 group sessions; 2 celebratory parties	[43]	Not assessed
**Parent–Child** **Interaction** **Therapy with** **Parent Emotion** **Coaching** **(PCIT-ECo)**	Integrates parent emotion coaching skills and parents’ implementation of these ER abilities during live parent–child interactions Reduces focus on direct child instruction in emotion identification Coaches caregivers in emotion socialization and modeling prosocial ER strategies	3–7 years	14+ sessions	[44]	Primary
**Parenting Your** **Hyperactive** **Preschooler**	Behavioral parent training program Emotion socialization strategies to enhance ER and to reduce ADHD symptoms	3–6 years	14 sessions	[45]	Primary
**Behavioral Parent** **Training (BPT)** **Targeting ED**	ED-focused psychoeducation to help parents understand their child’s dysregulated emotional responses Emphasizes aligning parenting strategies with children’s emotional states Strengthens caregivers’ emotion coaching and de-escalation skills	6–11 years	6 group sessions	[46]	Primary
**Managing Frustration for Children (MFC)**	Psychoeducation about ER Focuses directly on improving children’s ER skills (e.g., frustration tolerance, dysregulated emotion reactivity, problem solving)	9–12 years	12 group sessions	[47]	Primary
**Emotion** **Regulation Skills** **(ER-SKILLS)**	Integrates CBT, DBT, and ACT to target ED in adolescents with ADHD Focuses on emotion awareness, emotion clarity, acceptance of emotions, ER strategies, flexibility, and goal setting	13–17 years	8 sessions	[48]	Primary
**Regulating** **Emotions Like An eXpert** **(RELAX)**	Psychoeducational and skills-based groups (i.e., parent, child, combined parent/child) Caregivers are provided with ER and communication skills a week prior to their adolescents Simultaneously teach and practice conflict management strategies	11–16 years	8 group sessions + 2 Boosters	[30,49]	Primary

Note: ER = emotion regulation; ED = emotion dysregulation; ADHD = attention-deficit/hyperactivity disorder.

## Data Availability

No new data were created or analyzed in this study.
